# The Center for the Study and Support of Caregivers - Clinical Activities

**DOI:** 10.1093/geroni/igaf122.179

**Published:** 2025-12-31

**Authors:** Veerawat Phongtankuel

**Affiliations:** Weill Cornell Medicine, New York, New York, United States

## Abstract

Many family caregivers are untrained and often feel ill-prepared to take on caregiving tasks. Our clinical program focuses on developing strategies to assess, support, and educate our patients’ caregivers. In our geriatric outpatient practice at the Center on Aging, we have 10,000 patient visits annually. Approximately 50% of these visits include either paid or family caregivers who accompany the patients. We will use validated caregiver assessment tools developed by research colleagues and implement them in the practice. Based on the results of these assessments, we will develop educational and support resources for family caregivers that will be available online and in person so that isolated caregivers can be reached. These programs include a curriculum covering proper techniques for lifting and transferring patients to avoid back strain, feeding patients with swallowing difficulties, and positioning patients in beds and chairs to avoid bedsores. These resources will also be customized to educate the caregiver on the care recipient’s illness and specific care needs. The curriculum will also cover counseling on caregiver stress. This will include strategies for finding relief and managing stress by learning how to ask for help, involving other family members in caregiving tasks, and participating in support groups. These practices are essential for maintaining their own health and well-being. The clinical program will train caregivers wherever we render care, whether it be in the inpatient, outpatient, or home setting.

